# Clinical parameters and inflammatory biomarkers among patients with multibracket appliances: a prospective clinical trial

**DOI:** 10.1186/s12903-024-03995-3

**Published:** 2024-03-05

**Authors:** Priscila Ferrari Peron, Heinrich Wehrbein, Ambili Mundethu, Irene Schmidtmann, Christina Erbe

**Affiliations:** 1 Department of Orthodontics and Dentofacial Orthopedics, University Medical Center of the Johannes Gutenberg University, Augustusplatz 2, 55131 Mainz, Germany; 2 Institute of Medical Biostatistics, Epidemiology and Informatics, University Medical Center of the Johannes Gutenberg University, Obere Zahlbacher Str. 69, 55131 Mainz, Germany

**Keywords:** Dental plaque, Gingivitis, MMP8, aMMP8, Orthodontics

## Abstract

**Background:**

Aim of the presented study was to investigate changes in clinical parameters and active matrix metalloproteinase-8 (aMMP-8) levels in gingival crevicular fluid of patients before and during treatment with multibrackets appliances.

**Methods:**

Fifty-five adolescents scheduled for the treatment were included. Clinical parameters and subgingival samples were obtained at six time points: 1 week before appliance insertion (T0), 3 (T1), 6 (T2) weeks, 3 (T3), 6 (T4) months, and 1 year (T5) after that. Gingival index and plaque index were assessed to evaluated changes on the clinical status. Subgingival samples were collected to analyze changes in aMMP-8.

**Results:**

Scores for gingival and plaque index increased after bracket insertion. The gingival index increased from T2 (*p* < 0.05) until T5 (*p* < 0.0001). Plaque index also increased, reaching its maximum peak at T3 (*p* < 0.05). Moreover, an increase of aMMP-8 levels (*p* < 0.05) was noted. There was no significant between upper and lower jaws.

**Conclusions:**

Treatment with multibracket appliances in adolescents favors dental plaque accumulation and may transitionally increase gingival and plaque index and aMMP-8 levels leading to gingival inflammation, even 1 year after therapy began.

**Trial registration:**

This study was approved by the Ethics Committee of the dental medical association Rheiland-Pfalz, Germany (process no. 837.340.12 (8441-F)), and followed the guidelines of Good Clinical Practices.

## Background

Dental and skeletal malocclusions can have a negative impact on quality of life by interfering with the patient´s aesthetics, social interaction, and psychological well-being [1–5]. Moreover, it can affect functions of the stomatognathic system such as breathing, chewing, and swallowing. Due to these reasons, the malocclusion should be treated [4, 6–8].

Orthodontic therapy with multibracket appliance (MBA) is a widely used method for the treatment of malocclusions. However, the components of this appliance such as brackets, arches, ligaments, and tubes make oral hygiene difficult, affecting oral health by the increased accumulation of biofilm around the retentive structures [9, 10].

Thus, the high number of retention surfaces for biofilm together with poor oral hygiene can contribute to the development of white spot lesion [11–13], gingivitis [14–17], or even periodontal attachment loss [18–22]. Furthermore, it can lead to pathogenic bacterial colonization [23–28] and increased levels of matrix metalloproteinases (MMPs) found in gingival crevicular fluid (GCF), salivary fluid, and gingival tissues, triggering a periodontal disease [29, 30].

MMPs are proteolytic enzymes involved in the degradation and remodeling process of the extracellular matrix, both in physiological and pathological situations. MMP8 is considered the most significant collagenase found in CGF and is present in an active and latent forms. In its activated form, aMMP-8 decomposes periodontal tissue collagen leading to alveolar bone destruction [31–33].

Studies have shown that MMP8 plays an important role in the periodontal remodeling process during orthodontic movement [34–36]. MMP8 levels in GCF were found elevated after 4–8 h of orthodontic force application, this suggests that cells of periodontum are upregulated to express MMP-8, and its enhancement and activation indicates periodontal remodeling due to orthodontic force [37].

Interestingly, aMMP-8 concentration assessment in GCF or salivary fluid allows much earlier, non-invasive and more objective method to diagnose acute inflammatory events prior to clinical manifestations. Because of that, it has been used as an inflammatory and prevention biomarker in periodontal disease diagnosis [30, 38–51] and also to detect peri-implantitis lesions [42, 49, 51–56].

Traditional periodontal diagnostic indices such as clinical attachment level, bleeding on probing, GI [14, 57, 58], PI [59–62] or x-rays are only visible after the presence of inflammation or biofilm formation, or even after the presence of partially irreversible periodontal damage.

Since MBA treatment can negatively affect patients' oral health, the present study aimed to evaluate clinical aspects, using GI and PI, and levels of aMMP8 in GCF of MBA patients at different time points up to 1 year after MBA placement.

## Materials and methods

### Subjects

This prospective study was conducted at the Department of Orthodontics and Dentofacial Orthopedics, University Medical Center of the Johannes Gutenberg, Mainz, Germany. From a clinic´s currently patients list, 80 patients were blindly selected and asked by the study examiner if they wished to participate in this study either by telephone or personally when the patient came to the clinic for the first consultation. Fifty-five subjects, 30 females and 25 males aged between 12 to 17 years (mean age 13.81 ± 1.3) were then included in the study, which was performed between August 2013 and April 2017.

Inclusion criteria were a malocclusion with an indication for therapy with fixed orthodontic appliance in the upper jaw (UPJ) and lower jaw (LOJ); good general and periodontal health; a minimum of 16 natural teeth, including 8 anterior teeth. Subjects were exclude if they had a previous orthodontic treatment; more than 3 carious defects; periodontal disease; antibiotic intake or a professional dental cleaning two weeks before study start; patients with any kind of syndrome (Down-, Crouzon-, Apert-, Goldenhar-, Marfan-, Franchescetti-, Pierre-Robin-Syndrome); craniofacial anomalies such as cleft lip and palate; diabetes mellitus; allergies to dyes/colorants; pregnancy. All volunteers and their guardians were informed about the study procedures and signed a declaration of consent prior to participation.

### Overall study design

The entire study consisted of six time points (T): Baseline (T0): 1 week before bracket bonding (BB); T1: 3 weeks, T2: 6 weeks, T3: 3 months, T4: 6 months and T5: 1 year after BB. The subjects were instructed to brush their teeth before 8:00 AM on the study day and also not to eat or drink (except water) 2 h before the visit. Table 1 summarizes the overall study design.

**Table 1:** Study schedule by procedures according to different time points

Study Plan	T0	BB	T1	T2	T3	T4	T5
Informed Consent	X						
Medical History	X						
Inclusion / Exclusion Criteria	X						
Continuance Criteria			X	X	X	X	X
Bracket Bonding in UPJ and LOJ		X					
Oral Tissue Examination	X		X	X	X	X	X
Gingival Index	X		X	X	X	X	X
Plaque Index	X		X	X	X	X	X
GCF-Sample	X		X	X	X	X	X
Professional teeth cleaning		X				X	X
Oral Hygiene Instructions / Aid Distribution		X				X	X
Receipt Elmex Gelée		X				X	X
General Comments	X		X	X	X	X	X
Adverse Events			X	X	X	X	X

TO 1 week before bracket bonding, BB Bracket Bonding, T1 3 weeks after bracket bonding, T2 6 weeks after bracket bonding, T3 3 months after bracket bonding, T4 6 months after bracket bonding, T5 1 year after bracket bonding

At each visit a visual examination was performed to inspect the oral cavity. Teeth, gingival, palate, labial mucosa, tongue, mouth floor, and lips were examined and abnormal findings were noted. Afterwards, GI and PI were conducted. Subsequently, subgingival samples were collected.

BB occurred one week after T0. A professional dental cleaning was performed and conventional metallic brackets—nickel-free, system-slot 0.022″ (Micro Sprint Brackets—Forestadent®; Pforzheim, Germany) were bonded on the buccal teeth surfaces (except molars) in UPJ and LOJ with Transbond XT (3 M ESPE).

### Clinical procedures

#### Gingival index

The gingival color assessment, consistency, inflammation, and bleeding on probing was performed using the GI according to Löe and Silness [14]. All teeth except the molars were assessed. A modification was made to the GI regarding the number of observed areas, and instead of four gingival units (buccal, lingual, mesial and distal), each tooth was divided into six gingival areas: distobuccal, buccal, mesiobuccal, mesiolingual (or mesiopalatinal), lingual (or palatinal) and distolingual (or distopalatinal). Teeth and gingiva were gently dried with air before scoring to provide proper visibility. Then, without pressure, the periodontal probe tip was inserted about 1 mm into the gingival margin. Each of tooth surfaces received a score: 0: normal gingiva; 1: Mild inflammation – slight change in color, slight edema; no bleeding on probing; 2: Moderate inflammation – redness, edema, and glazing; bleeding on probing; 3: Moderate inflammation – redness, edema, and glazing; bleeding on probing. An index for the entire mouth was determined by dividing the total score by the number of surfaces examined.

#### Plaque index

The Turesky Modification of the Quigley-Hein PI [63] was performed by an experienced examiner to assess the plaque level on the buccal and lingual/palatinal surfaces of all teeth, except molars, crowns, and surfaces with cervical restoration. All teeth were stained using a foam pellet (Erkodent®, Pfalzgrafenweiler, Germany) and a plaque disclosing agent (Mira-2-Ton, Hager Werken, Duisburg, Germany). Subjects rinsed the mouth thoroughly with water and supragingival plaque was scored using a 0–5 Scale: 0: absence of plaque; 1: isolated plaque spots on the cervical margin; 2: a slim continuous layer of plaque (up to 1 mm) at the cervical margin; 3: a layer of plaque thicker than 1 mm; 4: plaque covering at least one-third of the side of the crowm of the tooth; 5: plaque covering two-thirds of the side of the crowm of the tooth [63]. To calculate whole mouth plaque level, the total score was divided by the number of examined teeth.

#### Subgingival samples – concentration of aMMP-8

Subgingival samples were taken after the participants had brushed their teeth’s to remove supragingival plaque, using a sterilized paper strip (GCF collection Strips – dentognostics GmBH; Jena, Germany) from the distal approximal space of the Ramfjord teeth [16, 12, 24, 36, 32, 44] [64, 65]. If a Ramfjord tooth was missing, a substitute tooth (teeth number: 17, 11, 25, 37, 31, 45) was chosen [66].The area was gently dried and with cotton rolls (Roeko-Luna; Coltène-Whaledent Gmbh, Langenau, Germany) isolated from oral fluid to avoid contamination. GCF samples collected as pool from the UPJ and the LOJ. All samples were send to Bioscientia Laboratory (Institut für Medizinische Diagnostic GmbH, Berlin) and a quantitatively analyzed for aMMP-8 using an enzyme-linked immunosorbent assay (ELISA—dentoELISA aMMP-8, dentognostics GmbH, Jena, Germany) was at these lab performed. The degree values of aMMP-8 levels (nanograms per milliliter (ng/ml)) with a cut-off 20 ng/ml for periodontitis were as following: ≤ 10 ng/ml: healthy; < 20 ng/ml: slow rate of progression; ≥ 20 ng/ml: moderate rate of progression [42, 67–69].

#### Statistical analysis

All statistical analyses were performed using SPSS (IBM® SPSS® Statistics, Version 23, IBM Corporation, Armonk, USA, © 1989, 2015). Mean value, standard deviation, interquartile distance, and median were determined for the assessment and evaluation of the GI, PI, and aMMP-8 levels values at the respective time point. Differences between UPJ and LOJ were assessed using paired t-tests comparing values at follow-up visits to baseline values. The significance level was chosen as α = 0.05. As numerous comparisons were performed and focus was on detecting possible changes and associations no formal adjustment for multiple testing was performed. Therefore, only the local significance level was controlled and the probability of obtaining at least one false positive result is substantially higher than 5%.

## Results

### Subjects

Fifty five adolescents (45.5% male and 54.5% female), 98.2% Caucasians and 1.8% Asian, were screened and 50 completed the roll study. One subject had the MBA removed before 1 year of therapy and missed the last time point. Two subjects moved to another city and therefore could no longer participate in this trial. And two other subjects showed a lack of compliance and both were excluded after the 5th time point.

### GI, PI values and aMMP8 levels

GI, PI values and aMMP8 levels are presented first as an index in UPJ and LOJ at different time points, and second as a comparison between UPJ and LOJ.

**Fig. 1:**
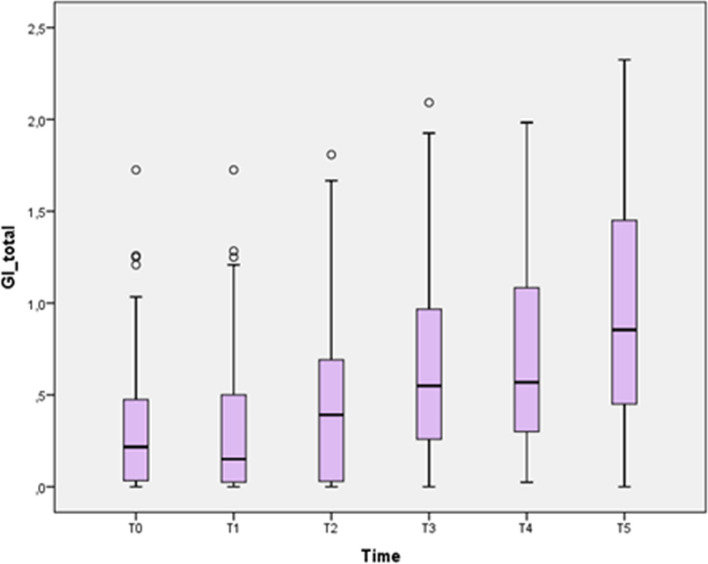
Boxplot diagram showing GI for the entire UPJ and LOJ at different time points. TO: 1 week before bracket bonding; T1: 3 weeks after bracket bonding; T2: 6 weeks after bracket bonding; T3: 3 months after bracket bonding; T4: 6 months after bracket bonding; T5: 1 year after bracket bonding GI Scores: 0: normal gingiva; 1: Mild inflammation; 2: Moderate inflammation; 2,5: severe inflammation

GI total gradually increased (*p* < 0.0001) after T2 until T5, when it had reached its maximum peak signaling a tendency to gingival inflammation even 1 year after treatment began (Fig. 1).

**Fig. 2:**
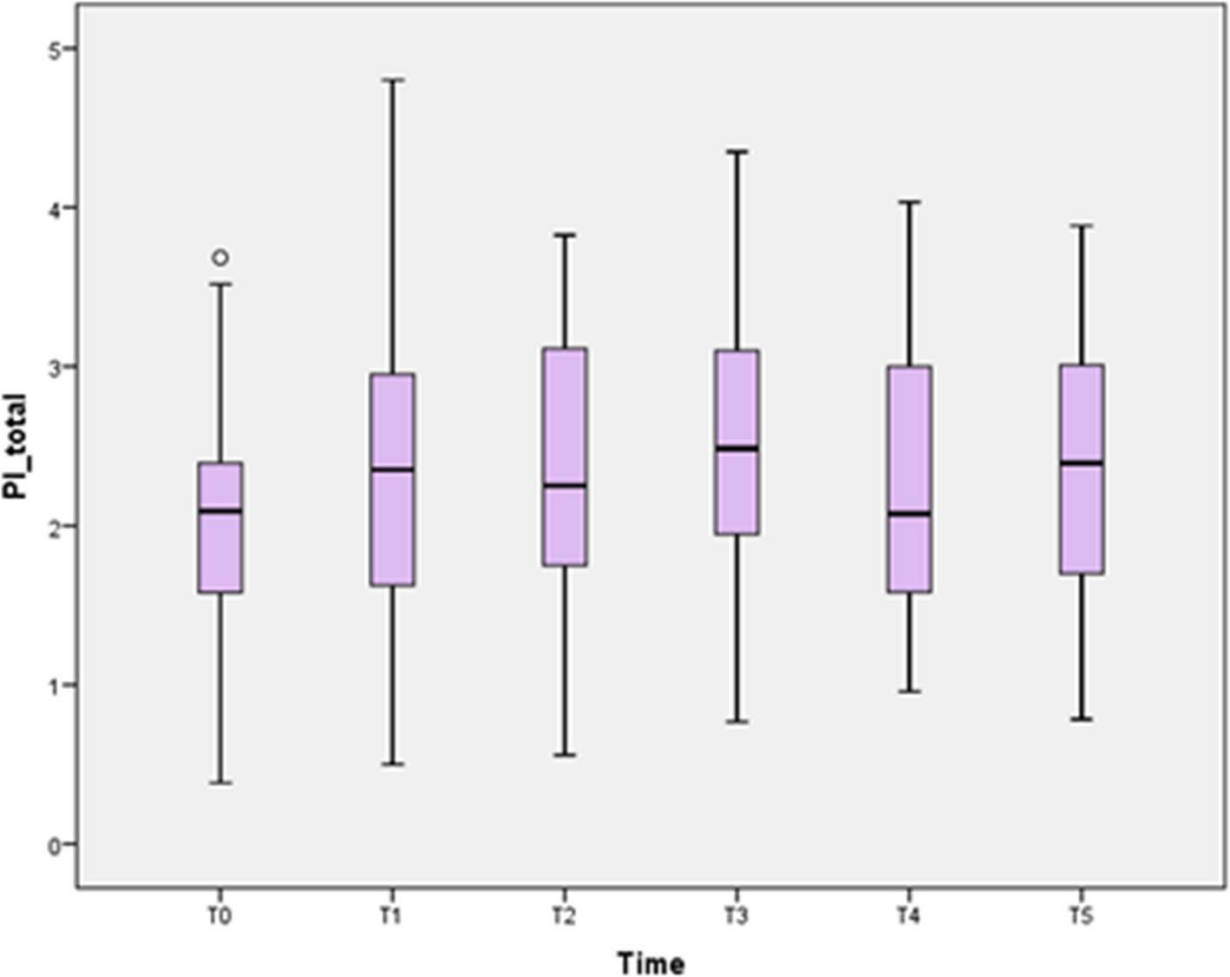
Boxplot diagram showing the PI for the UPJ and LOJ at different time points.TO: 1 week before bracket bonding; T1: 3 weeks after bracket bonding; T2: 6 weeks after bracket bonding; T3: 3 months after bracket bonding; T4: 6 months after bracket bonding; T5: 1 year after bracket bonding. PI Scores: 0: no plaque; 1: single plaque areas; 2: plaque lines; 3: plaque extension up to 1/3 of tooth surface; 4 plaque extension up to 2/3 of tooth; 5: plaque extension more than 2/3 of the tooth surface

PI total values as well GI values continued to increase after appliance installation, until T3, when it reached its maximum peak (*p* < 0.05). Then, there was a small decrease at T4 followed by an increase at T5 (*p* < 0.05) (Fig. 2).

**Fig. 3:**
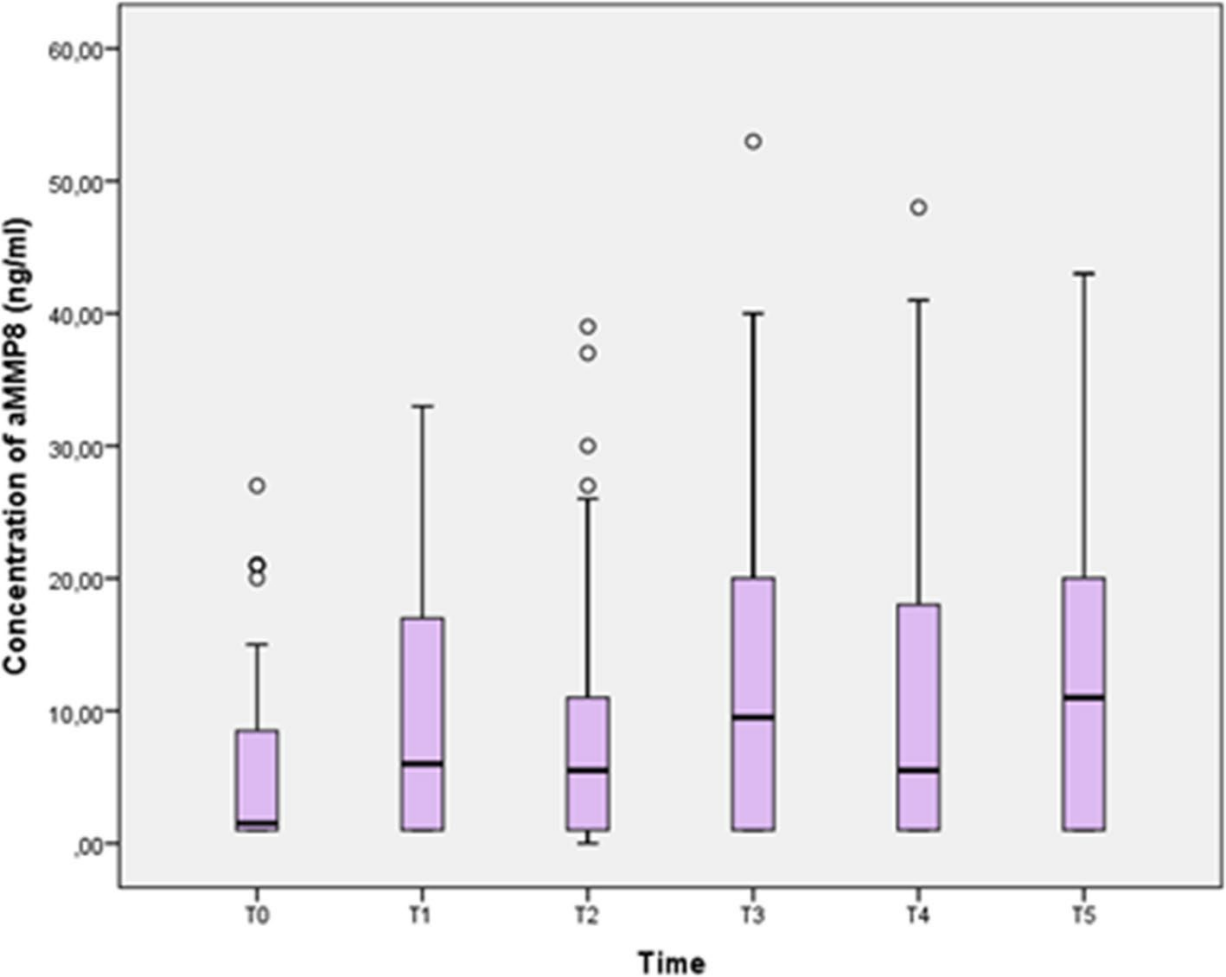
Boxplot showing the aMMP-8 levels in UPJ and LOJ at different time points. TO: 1 week before bracket bonding; T1: 3 weeks after bracket bonding; T2: 6 weeks after bracket bonding; T3: 3 months after bracket bonding; T4: 6 months after bracket bonding; T5: 1 year after bracket bonding aMMP8 Scores: ≤ 10 ng/ml: healthy; < 20 ng/ml: slow rate of progression; ≥ 20 ng/ml: moderate rate of progression

The levels of aMMP-8 in GCF increased significantly at T1, T3, and T5, when reached its maximum peak, signalizing a low degree of inflammation (Fig. 3). Table 2 summarizes the total GI, PI, and the concentration of aMMP8 at different appointments.

**Table 2:** Statistical parameters of the total GI, PI and aMMP8 levels (ng/ml) at different time points

Time Point / "*n*"	total	Lower Quartile	MV (SD)	Median	Upper Quartile	Min.-Max	Mean changes (SD)	*p*-value
**T0**	**GI**	0.03	0.34 (0.39)	0.22	0.48	0 – 1.73	NA	NA
(*n*** = **55)	**PI**	1.57	2.03 (0.68)	2.09	2.40	0 – 4	NA	NA
	**aMMP8**	1.00	5.29 (7.08)	1.50	9.00	0 – 27	NA	NA
**T1**	**GI**	0.03	0.32 (0.40)	0.15	0.50	0 – 1.73	-0.01 (0.19)	0.6718
(*n* = 53)	**PI**	1.57	2.29 (0.92)	2.35	2.96	0 – 5	0.27 (0.86)	0.0279*
	**aMMP8**	1.00	10.21 (9.95)	6.00	18.00	0 – 33	4.16 (9.15)	0.0017*
**T2**	**GI**	0.03	0.48 (0.48)	0.39	0.73	0 – 1.81	0.14 (0.36)	0.0063*
(*n* = 55)	**PI**	1.75	2.38 (0.87)	2.25	3.12	1 – 4	0.35 (0.89)	0.0055*
	**aMMP8**	1.00	8.55 (9.54)	5.50	11.00	0 – 39	2.63 (10.45)	0.0677
**T3**	**GI**	0.25	0.68 (0.53)	0.55	1.00	0 – 2.09	0.34 (0.45)	0.0001^**^
(*n* = 55)	**PI**	1.93	2.48 (0.82)	2.48	3.11	1 – 4	0.44 (0.80)	0.0002*
	**aMMP8**	1.00	12.26 (12.8)	9.50	20.00	0 – 53	6.34 (11.87)	0.0002*
**T4**	**GI**	0.30	0.72 (0.54)	0.57	1.08	0 – 1.98	0.38 (0.49)	0.0001^**^
(*n* = 54)	**PI**	1.58	2.23 (0.80)	2.08	3.00	1 – 4	0.20 (0.75)	0.0533
	**aMMP8**	1.00	10.43 (11.7)	5.50	18.00	0 – 48	4.42 (13.43)	0.0192*
**T5**	**GI**	0.45	0.95 (0.60)	0.85	1.45	0 – 2.33	0.62 (0.62)	0.0001^**^
(*n* = 50)	**PI**	1.69	2.39 (0.83)	2.39	3.03	1 – 4	0.35 (0.96)	0.0126*
	**aMMP8**	1.00	13.05 (11.9)	11.0	20.00	0 – 43	7.01 (12.04)	0.0001*

*SD* Standard deviation, *MV* mean value; T0 Baseline 1 week before MBA, T1 3 weeks after MBA, T2 6 weeks after MBA, T3 3 months after MBA, T4 6 months after MBA, T5 1 year after MBA

* p < 0.05; ** p < 0.0001

There was no statistically significant difference between UPJ and LOJ regarding GI, PI, and aMMP8. Table 3 provides additional details about the comparison these parameters in UPJ and LOJ.

**Table 3:** GI; PI; aMMP8 levels (ng/ml) – Comparison between upper and lower jaw

Time Point	GI / PI / aMMp8	**UPJ** MV (SD)	**LOJ** MV (SD)	UPJ *vs* LOJ MV (SD)	*p*-value
Baseline (T0)	**GI**	0.36 (0.59)	0.32 (0.36)	0.04 (0.58)	0.5895
**(*N* = 55)**	**PI**	2.02 (0.77)	2.05 (0.71)	-0.03 (0.55)	0.6981
	**aMMP-8**	3.03 (4.18)	2.90 (3.42)	0.13 (2.84)	0.7409
Time 1 (T1)	**GI**	0.35 (0.58)	0.30 (0.38)	0.05 (0.58)	0.4949
**(*N* = 53)**	**PI**	2.23 (1.00)	2.34 (0.94)	-0.11 (0.58)	0.1905
	**aMMP-8**	5.37 (5.72)	4.85 (5.09)	0.52 (4.28)	0.3814
Time 2 (T2)	**GI**	0.50 (0.67)	0.45 (0.47)	0.05 (0.65)	0.5642
**(*N* = 55)**	**PI**	2.33 (0.95)	2.43 (0.95)	-0.10 (0.74)	0.3264
	**aMMP-8**	4.33 (5.21)	4.23 (5.06)	0.10 (3.76)	0.8445
Time 3 (T3)	**GI**	0.67 (0.72)	0.69 (0.55)	-0.02 (0.69)	0.8216
**(*N* = 55)**	**PI**	2.38 (0.93)	2.57 (0.91)	-0.19 (0.83)	0.1022
	**aMMP-8**	6.35 (7.19)	5.91 (6.81)	0.45 (5.67)	0.5623
Time 4 (T4)	**GI**	0.68 (0.70)	0.77 (0.65)	-0.10 (0.82)	0.3930
**(*N* = 54)**	**PI**	2.19 (0.97)	2.28 (0.79)	-0.09 (0.75)	0.3897
	**aMMP-8**	5.32 (6.14)	5.11 (6.94)	0.21 (5.89)	0.7916
Time 5 (T5)	**GI**	0.86 (0.68)	1.05 (0.75)	-0.19 (0.78)	0.0869
**(*N* = 50)**	**PI**	2.29 (0.92)	2.47 (0.88)	-0.19 (0.70)	0.0631
	**aMMP-8**	6.32 (6.10)	6.73 (6.58)	-0.41 (4.32)	0.5048

*SD* Standard deviation, *MV* mean value, T0 Baseline 1 week before MBA, T1 3 weeks after MBA, T2 6 weeks after MBA, T3 3 months after MBA, T4 6 months after MBA, T5 1 year after MBA

For statistical analysis was used the program SPSS (IBM® SPSS® Statistics, Version 23, IBM Corporation, Armonk, USA, © 1989, 2015). Changes of GI, PI, and aMMP-8 over time and differences between upper and lower jaw were assessed using paired t-tests comparing values at follow-up visits to baseline values. The significance level was chosen as α = 0.05. As numerous comparisons were performed and the focus was on detecting possible changes and associations no formal adjustment for multiple testing was performed. Therefore, only the local significance level was controlled and the probability of obtaining at least one false positive result is substantially higher than 5%.

## Discussion

Periodontal inflammation and caries are the main concern during fixed appliance treatment. The present study reports a significant increase in GI and PI values as well as aMMP8 levels in adolescents after MBA insertion, even up to 1 year after treatment begin. Thereby suggesting that plaque accumulation could be the causative factor for the gingivitis reported in these patients. Increased GI and PI values in the first therapy months may be due to patient's unfamiliarity with the appliance and difficulty in maintaining a proper oral hygiene. The reduction noted in these values after 6 months of treatment supports the fact that dental alignment allows the patient to achieve a good gingival condition around previously misaligned teeth [70]. However, the long orthodontic treatment duration can lead to a lack of patient motivation to perform good oral hygiene, justifying the observed increase in PI values 1 year post fixed appliance insertion.

Our results were in accordance with Naranjo et al. [17] and Guo et al. [10], regarding the elevated GI and PI values 3 months after MBA insertion. Ristic et al. [27, 28] also reported an increase after 3 months of therapy followed by a decrease 6 months later. Liu et al. [71] reported a significant decrease of these indices after 1 week of appliance removal, returning to pre-treatment values. Kim et al. [26] also related a decrease in GI and PI values 6 months after therapy end. A systematic review conducted by Cerroni et al. suggests that there is moderate scientific evidence that a fixed appliance negatively influences periodontal status [72]. On the other hand, Gomes and co-workers [73] stated that orthodontic appliances use is not necessarily related to periodontal conditions aggravation, but rather to each person´s susceptibility to periodontal disease.

Nonetheless, the majority published studies in the literature have a patient follow-up up to 3 or 6 months after brackets placement or post-appliance removal and with a small sample size. In contrast, our study accompanied 55 patients until 6 months and 50 subjects up to 1 year of MBA treatment. More long-term studies are necessary to be conduct on a wider sample size containing a control group to evaluate MBA effects on periodontium after years of treatment. Hence, our findings endorse previous reports showing an existing correlation between GI and PI values before and during orthodontic treatment. Accordingly, it can be suggested that plaque accumulation favored by brackets and archwires can cause gingival inflammation. Moreover, it is implied that MBA induces gingival inflammation without damaging the dental support tissues.

Though, a present study´s limitation is regarding GI and PI analysis. Both indices express the total buccal and palatinal/lingual surfaces sum values. It would be interesting to compare these two surfaces, once the brackets were bonded on the buccal teeth surface. A second point which should be considered is GI and PI distribution between anterior and posterior segments.

Regarding to aMMP8, the majority studies found in the literature associate the high presence of MMP-8 in GCF of orthodontic patients with periodontal ligament remodeling process [74, 75] and with pain mentioned by some patients during the first hours/days after appliance placement [76]. Surlin et al. [34] reported an increase in MMP-8 concentration in the first 4–8 h after orthodontic appliance placement followed by a decrease to initial levels. Some subjects developed gingival overgrowth (GO) during orthodontic treatment even in bacterial plaque absence. Interestingly, in these patients MMP-8 levels continued to increase until GO appearance. Furthermore, some patients presented GO in combination with inflammation and in these cases, MMP-8 concentration was higher than in GO cases without inflammation. In this way, the authors suggest that MMP-8 may be a possible biomarker for GO beginning [51].

Our study´s novelty was the use of aMMP-8 as periodontal biomarker in patients undergoing orthodontic treatment. It provides original evidence that 3 weeks after brackets placement there was a significant increase of aMMP-8 levels, which remained elevated even 1 year after treatment began, suggesting an inflammations tendency. aMMP8 high rates evidenced in this study agree with the high GI and PI scores.

## Conclusion

In summary, it can be concluded that the therapy with MBA may transitionally increase gingivitis, plaque accumulation, and aMMP-8 levels even 1 year after the beginning of therapy, occasionally leading to gingival inflammation but without destruction of periodontal supporting tissue. No significant differences were found between UPJ and LOJ values.

Since changes in clinical parameters and GCF increase the risk of periodontal tissue inflammation, proper oral hygiene instructions should be given to orthodontic patients in order to provide good oral hygiene, constant motivation, and continuous plaque control during the entire treatment. Long-term studies are needed to explore the impact of bacterial colonization on periodontal conditions and clinical aspects during the years of orthodontic treatment with fixed appliance and after its removal.

## Data Availability

The datasets used and/or analyzed during the current study may be available from the corresponding author on reasonable request.

## Abbreviations

MMPs: Matrix metalloproteinase
aMMP8: Active matrix metalloproteinase-8
MBA: Multibracket appliance
GCF: Gingival crevicular fluid
GI: Gingival index
PI: Plaque index
UPJ: Upper jaw
LOJ: Lower jaw
BB: Bracket bonding
T: Time points
